# Outcome of whole brain irradiation with a dose-escalated simultaneous-integrated boost in patients with multiple large and/or diffuse brain metastases: real live data and review of the literature

**DOI:** 10.1007/s12672-024-01176-w

**Published:** 2024-08-07

**Authors:** Linda Agolli, Luca Nicosia, Thomas Hilger, Gheorghe Iancu, Ann-Katrin Exeli, Bastian Eul, Tobias Struffert, Till Acker, Daniel Habermehl

**Affiliations:** 1 Department of Radiation Oncology, Justus-Liebig-University Giessen, Giessen-Marburg University Hospital, Klinikstraße 33, 35392 Giessen, Germany; 2 Advanced Radiation Oncology Department, Cancer Care Center, IRCCS Sacro Cuore Don Calabria Hospital, Verona, Italy; 3 Medical Physics, Radiation Oncology Radprax MVZ GMBH, Wuppertal, Germany; 4 Medical Physics, Department of Radiation Oncology, Giessen-Marburg University Hospital, Giessen, Germany; 5 Department of Internal Medicine, Justus-Liebig-University Giessen, Giessen-Marburg University Hospital, Giessen, Germany; 6 Justus-Liebig-University Giessen, Giessen-Marburg University Hospital, NeuroradiologyGiessen, Germany; 7 Justus-Liebig-University Giessen, Giessen-Marburg University Hospital, NeurorpathologyGiessen, Germany

## Abstract

**Background:**

We retrospectively investigate feasibility and safety of whole brain radiotherapy (WBRT) including a simultaneous-integrated boost technique (WBRT-SIB) in a cohort of patients with a very poor prognosis suffering from multiple and/or large brain metastases, unfavorable primary histology, poor performance status and/or symptomatic BMs.

**Materials and Methods:**

Thirty-five patients with high brain tumor burden, extracranial metastases and low life-expectancy were treated with WBRT-SIB mostly with 35-42 Gy/14 fractions. All metastases were boosted in patients with up to 12 BMs. In patients with > 12 BM, large and/or small metastases in critical brain regions were boosted up to a maximum of 12 SIB volumes.

**Results:**

The median number of BM was 8 (range 2–45) and the median BM diameter was 12 mm (range 4–90 mm). Fifteen (43%) patients had ≥ 10 BMs and 25 patients presented with a Karnofski index ≤ 80%. Primary tumor histology was NSCLC (n = 13), SCLC (n = 11), breast cancer (n = 7), melanoma (n = 2), other (n = 2). The median iPFS was not reached, and 12- and 18-months iPFS were 75% and 50%, respectively. Overall, seven patients had intracranial progression: two patients within the SIB and WBRT area, one patient only within the SIB region and four patients had new BMs in the WBRT volume alone. The median iPFS for non-SCLC patients was 17 months and the 12- and 18-month iPFS were 56.8% and 28.4%, respectively. There was no significant OS difference between SCLC-group and non-SCLC patients (p = 0.38). Overall, median OS was 8.7 months and 1-year OS was 25%. The treatment was generally well-tolerated with no observed cases of radionecrosis.

**Conclusion:**

Our WBRT-SIB approach involves a combination of whole brain radiotherapy and a simultaneous integrated boost to specific tumor volumes, and its effectiveness is compared with other treatment modalities in the literature. Further research, including prospective studies with larger patient cohorts, is necessary to validate and refine the findings.

## Introduction

The incidence rates of brain metastases (BM) in patients with solid tumor range from 7 to 14 persons per 100,000 population per year across population-based studies [1] and shows an increase over time due to improved survival, development of modern therapies and advancing diagnostic technologies [2].

The primary malignancies that mostly metastasize to the brain are lung, breast, esophageal, skin melanoma, colorectal, and kidney/renal pelvis [2]. Stereotactic radiotherapy (SRS) is widely used in the treatment of BM with high rates of local control [3]. Although, after SRS higher intracranial relapse rates have been reported compared to whole brain irradiation (WBRT) [4]. Moreover, SRS or fractionated stereotactic radiotherapy (FSRT) has been offered to selected patients in good clinical conditions, limited or controlled systemic disease and a limited number of BM, usually up to 10 [5]. Patients not suitable to SRS/FSRT with multiple (> 10 BMs) or large brain lesions, poor performance status and uncontrolled primary tumor are often selected for a WBRT approach or sometimes best supportive care (BSC) [6].

In the era of technological advances in radiation oncology, WBRT could be combined to SRS to the macroscopic BM or to sequential boost (SEB) by using an intensity modulated radiotherapy/volumetric modulated arc therapy (IMRT/VMAT) planning method [7] in patients with multiple BM to achieve durable intracranial control.

Recently, a few retrospective reports described good outcome in terms of local and intracranial control after WBRT + SRS or SEB, as well as after WBRT delivered with simultaneous integrated boost (SIB) [8–19]. In particular, survival benefits have been observed in the WBRT-SIB groups compared to other boost delivery techniques combined to WBRT in patients with diffuse brain metastases. Also, WBRT-SIB associated to new generation brain effective target therapy showed promising results with acceptable toxicity profile [12, 13].

In the literature, there is a very poor evidence of brain irradiation in real world patients with more than 10 or large metastases, reduced clinical conditions, poor prognosis due to primary histology as SCLC or melanoma. However, this unfavorable category of patients presents with symptomatic disease, limited quality of life is often considered only for 3 dimensional WBRT or BSC. Though, features of WBRT and doses remain an open question.

We retrospectively investigate feasibility and safety of WBRT-SIB in an unfavorable series of patients with multiple and or large brain metastases, unfavorable primary histology, poor performance status and/or symptomatic disease. To our knowledge, this is the first report including WBRT-SIB as a brain irradiation option in unfavorable patients’ group with high brain tumor burden, extracranial metastases and low life-expectancy.

## Methods

### Patients

This retrospective study included 35 patients with BM treated with WBRT-SIB from June 2021 to September 2023. Among the analyzed patients, all completed radiotherapy. Only one patient died before starting treatment and was excluded from the analysis. We selected a series of unfavorable patients with the following characteristics: large brain metastases ≥ 4 cm and/or diffuse brain metastases ≥ 10 intracranial lesions including those in the leptomeningeal area and brainstem metastases, active extracranial disease, small-cell lung cancer (SCLC) primary tumor histology and melanoma, neurological symptoms at BMs presentation and sometimes in poor clinical condition, not suitable to SRS. Further inclusion criteria for the analysis were histological confirmed tumor diagnosis, contrast agent enhanced magnetic resonance imaging (MRI), Karnofsky Index (KI) score ≥ 50%, expected survival time ≥ 3 months, completed treatment and follow-up information until at least up to 3 months after RT, patients with large resected metastatic cavities ≥ 4 cm.

Patients’ information including clinical records, imaging and follow up data were retrospectively reviewed from electronic medical records. The study was performed in accordance with the Declaration of Helsinki Version 2013. All the patients signed the informed consent and the study was approved from our internal review board of Justus-Liebig University of Giessen (ethic approval AZ 211/23 – Ethik-Kommission des Fachbereiches Medizin).

### Treatment

**Fig. 1:**
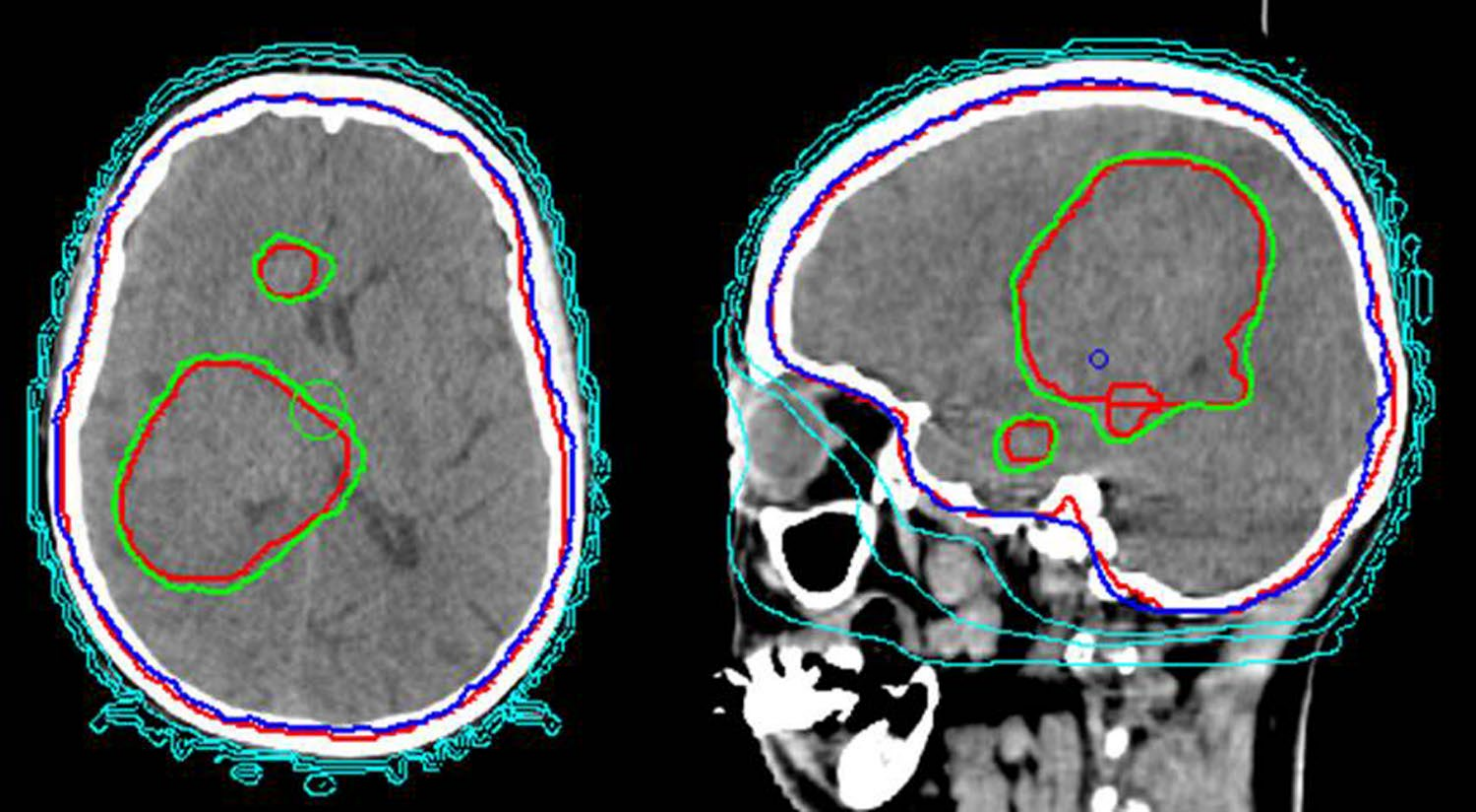
Example of a treatment plan using whole brain radiotherapy with simultaneous integrated boost (WBRT-SIB) in axial and sagittal slice

All patients received a contrast-enhanced MRI with 1-mm-thick slices including a 3D-corrected MP-RAGE series up to 7–10 days before treatment. This imaging was fused with the planning CT imaging for precise volume definition of brain metastases. All patients received a stereotactic fixation mask. The radiation dose for WBRT was mostly 35 Gy/14 fractions (daily dose 2,5 Gy) with a simultaneous boost of 37.8–42 Gy in 14 fractions (daily dose 2.67–3.0 Gy according to size and location). All metastases were boosted in patients with up to 12 BMs (see Fig. 1). In patients with > 12 BM, large and/or small metastases in critical brain regions were boosted up to a maximum of 12 SIB volumes. The clinical target volume (CTV) for WBRT was the entire brain. The planning target volume (PTV) was an isotropic expansion with a margin of 3 mm to the CTV (elective brain volume). The GTVs of the single lesions were delineated based on contrast enhancement on T1-MRI. The SIB-PTVs were generated by adding a 3D isotropic margin of 2 mm to the GTV. Spinal cord, eyes, lenses, optic nerves, optic chiasm, brainstem, cochlea, lacrimal glands and parotid gland were defined as organs at risk. A hippocampus-sparing approach was considered by an absence of BMs in the hippocampus region and was possible only in 3 patients; both hippocampi were contoured as OAR. No sparing was performed in the other patients because of metastases located also in the hippocampus region or due to a high brain tumor burden.

The treatment was planned using volumetric modulated arc therapy (VMAT) with 2–3 coplanar full arcs and 6MV.

### Statistical analysis

The follow-up after treatment was performed with brain MRI at nearly 4–6 weeks after WBRT-SIB, then every 3 months after radiotherapy until death or loss during follow-up. The response was evaluated by experienced radiologists according to the RECIST criteria. Toxicities associated with radiotherapy were evaluated according to the CTC AE Version 4.0 central nervous system toxicity criteria.

The primary endpoint was the intracranial progression free survival (iPFS) and local control. The secondary endpoints were intracranial response, pattern of intracranial progression, 6-months overall survival (OS), and tolerance with WBRT-SIB. The iPFS was defined as the time from RT start to intracranial progression confirmed in MRI or death. The overall survival (OS) was defined as the time from RT start to death or the last follow-up. Intracranial progression was defined as evidence of new intracranial BM on brain MRI or an increase of > 20% in the diameter of already present BM. Survival were calculated with the Kaplan Meier method and univariate analysis was performed with the log-rank test. The statistical analysis was performed using SPSS version 20.0 software (IBM, Chicago, IL). A p ≤ 0.05 indicated a significant correlation.

## Results

### Patients’ characteristics

Thirty-five (n = 35) patients with multiple/large BM were selected. The median number of BM was 8 (range 2–45), the median BM diameter was 12 mm (range 4–90 mm), and the median PTV boost volume was 17.7 cc (range 1.67–50.23). BM presentation was synchronous in 18 cases (51.4%) and metachronous in 17 patients (48.6%). Fifteen (43%) patients had ≥ 10 BM and 25 patients presented with KI ≤ 80%. Primary tumor histology was as follows: NSCLC (n = 13), SCLC (n = 11), breast cancer (n = 7), melanoma (n = 2), other (n = 2). Twenty-tree patients had also extracranial metastases at the time of BM diagnosis. See patients’ characteristics in Table 1.

**Table 1:** Clinical characteristics of the study population (n = 35)

Characteristics	Value or No. of Patients (%)
Age, years Median (range)	63 (42–90)
Gender Male Female	12 (34%) 23 (66%)
Karnofsky Index % 100 90 80 50–70	0 (0%) 15 (42.5%) 9 (25.5%) 11 (32%)
Symptoms before RT yes no	19 (54.2%) 16 (45.8%)
Neurocognitive impairment before RT yes no	14 (40%) 21 (60%)
Primary NSCLC SCLC Breast cancer Melanoma Others	13 (37%) 11 (32%) 7 (20%) 2 (5.5%) 2 (5.5%)
Brain metastases to primary Synchronous Metachronous	19 (54.2%) 16 (45.8%)
Extracranial metastases at the time of BM yes no	27 (77%) 8 (33%)
Site of brain metastases Supra-/infratentorial Only supratentorial Leptomeningeal Brainstem	10 (28.5%) 19 (54.5%) 3 (8.5%) 3 (8.5%)
Metastases/resection cavities (n) 1–3 4–9 ≥ 10 Range 1–45 BM	6 (17%) 14 (40%) 15 (43%)
MRI diameter (mm) Median (range)	12 (4–90)
PTV SIB (cc) Median (range)	17.67 (1.67–50.23)

RT: radiotherapy; NSCLC: non-small-cell lung cancer; SCLC: small cell lung cancer; DM: distant metastases; BM: brain metastases; PTV: planning tumor volume; SIB: simultaneous integrated boost; cc: cubic centimeter

Patients with limited and well-defined leptomeningeal metastases were also considered for WBRT-SIB. Nineteen patients presented with neurological symptoms at diagnosis of BM and/or 14 of them had neurocognitive impairment before RT. Baseline symptoms are reported in Table 2.

**Table 2:** Baseline symptoms and toxicity profile after treatment

Baseline symptoms toxicity	Grade CTC AE V.4		
Baseline	G1	G2	G3
Headache	4	2	0
Nausea	1	1	0
Vomiting	0	0	1
Dizziness	2	3	0
Diplopia	0	1	0
Ataxia	0	2	0
Seizure	0	1	0
Dysarthria/Dysphasia	0	1	0
Hemiparesis	0	0	1
Acute toxicity after WBRT-SIB	G1	G2	G3
Ataxia	1	0	0
Fatigue	5	5	0
Headache	1	5	0
Dizziness	2	0	0
Nausea	1	3	0
Vomiting	1	0	0
Dermatitis	3	0	0
Alopecia	8	4	0
Concentration impairment	1	0	0
Seizure	0	0	1
Late toxicity after WBRT-SIB	G1	G2	G3
Dermatitis	3	0	0
Hair loss	3	0	0
Leukoencephalopathy	0	2	0
Concentration/memory impairment	0	1	0

### Local control and survival

Local response of active BM al the last follow-up was as follows: five patients (14.5%) had a complete response, 14 (40%) had a mixed response, 9 (25.5%) had a partial response, and seven (20%) had a stable disease.

**Fig. 2:**
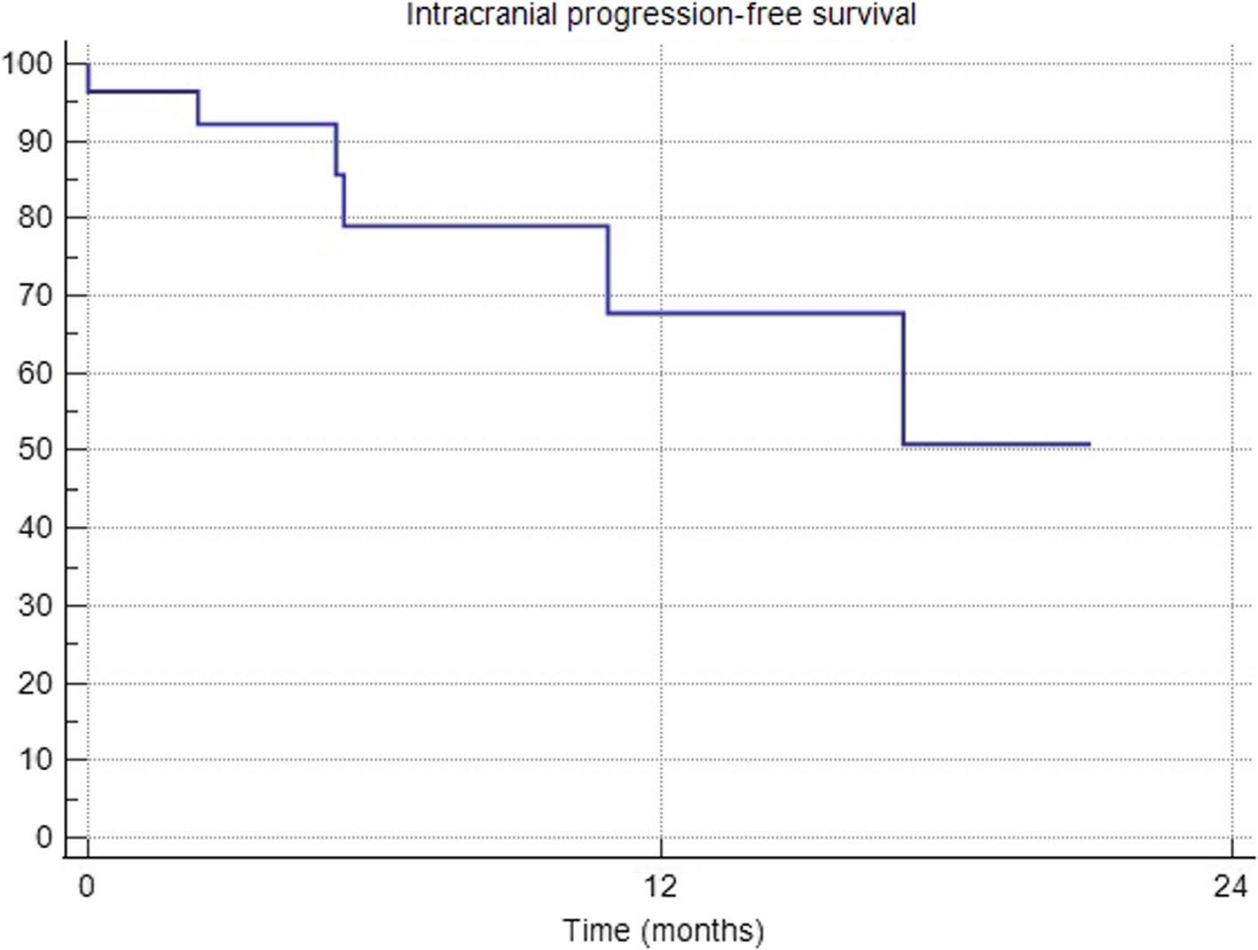
Intracranial progression-free survival (iPFS)

In the entire series, the 12- and 18-months local control of BM within the SIB area was 83%, respectively. The median iPFS was not reached, and 12- and 18-months iPFS were 75% and 50%, respectively (see Fig. 2). Overall, seven patients had intracranial progression at last follow up: 2 patients within the SIB and WBRT area, one patient only within the SIB region and 4 patients had new BMs in WBRT area alone. Local relapse occurred within the SIB area in 3 patients as follows: one patient showed a relapse of 2 BMs after previous CR, one patient had a relapse of a single BM after previous PR and underwent surgery, one had a relapse of only one BM and died two weeks after diagnosed BM recurrence due to systemic progression.

The median iPFS for non-SCLC patients was 17 months and the 12- and 18-month iPFS were 56.8% and 28.4%, respectively. There was no difference in iPFS between SCLC and non-SCLC patients (p = 0.17).

**Fig. 3:**
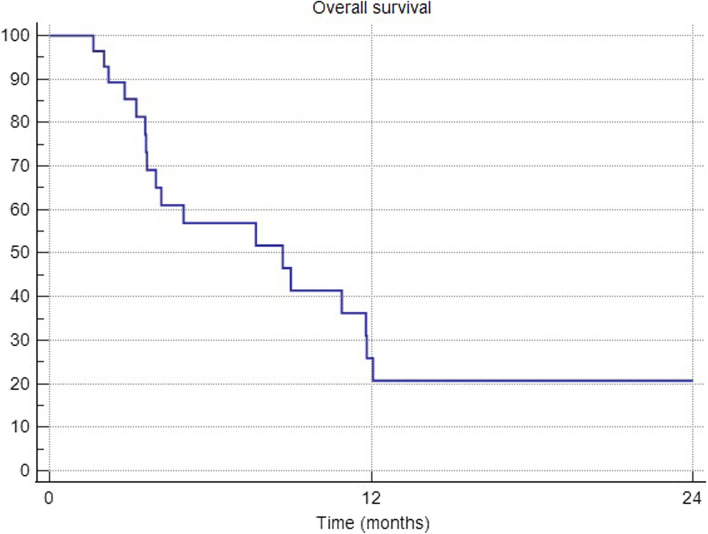
Overall survival (OS)

Overall, median OS was 8.7 months and 1-year OS was 25%. (see Fig. 3). The median OS for non-SCLC (any other primary tumor than SCLC) patients was five months and the 12-months OS was 27%. There was no significant OS difference between SCLC-group and non-SCLC patients (median 11.8 versus 5 months; p = 0.38).

At the last follow-up, death occurred in 20 patients due to systemic progression (15 patients; 75%), mixed systemic and intracranial progression in two cases (20%) and intracranial progression in one patient (5%). Overall, the median OS was 8.7 months (range 4–12), and the 1-year OS was 25.3%.

### Toxicity/symptoms

Among 35 treated patients, 14 (40%) had baseline neurological symptoms before WBRT + SIB. After treatment, 8 of these patients (57%) had symptom improvement during the follow up. Patients experienced multiple acute treatment-related symptoms as follows: grade 1 toxicity occurred in 17 cases (48.5%), grade 2 toxicity in 14 patients (40%), and grade 3 in two patients (5.7%). More in details, grade 1 toxicity was represented by hair loss (8; 22.8%), fatigue (5; 14.2%), headache (1; 2.8%), dizziness (2; 5.7%), dermatitis (3; 8.5%), nausea (1; 2.8%), vomiting (1; 2.8%), concentration impairment (1; 2.8%) and ataxia (1; 2.8%). Grade 2 toxicity occurred as follows: headache (5; 14.2%), fatigue (5; 14.2%), hair loss (4; 11.4%), and nausea (3; 8.5%). Only one patient developed grade 3 generalized seizure requiring hospitalization, although this symptomatic could be due also to metastatic brain disease itself. Late toxicity occurred in 8 patients (22.8%) and was represented by: grade 1 dermatitis (3; 8.5%), grade 1 hair loss (3; 8.5%), concentration/memory impairment (1; 2.8%), grade 2 leukoencephalopathy (2; 5.7%). No cases of radionecrosis were reported. Toxicities is reported in Table 2.

## Discussion

Stereotactic radiotherapy technique is widely used in the treatment of limited metastatic disease [20]. Cranial SRS/FSRS are effective treatment options in patients with a limited number of BM showing good local control rates [21, 22]. However, metastatic patients often present with multiple or large BM, poor clinical conditions, uncontrolled primary and tumor spread related symptomatic. Sometimes, this unfavorable category of patients is selected to WBRT or BSC [23, 24].

WBRT is used to treat multiple brain metastases addressing both visible and microscopic tumors and could help prevent the development of new metastases in other brain areas. The SIB component allows for the delivery of a higher radiation dose to specific tumor regions within the brain. This is often based on the size and location of the metastases.

In the literature, there are only retrospective series regarding WBRT-SIB in patients with intracranial metastatic disease (See Table 3). Overall, there are about > 400 treated patients in the past 5 years. Most of them were NSCLC patients; a few patients presented with breast cancer, melanoma, SCLC or a primary tumor in the GI tract [8, 13, 17, 22]. Mainly, WBRT-SIB was performed among patients with 1–4 BMs, a few of them had between 4 and 10 BMs and a very few patients presented with > 10 BMs. In the current study, unfavorable patients with large/multiple BMs and high brain tumor burden (median PTV SIB volume 18 cc) were considered for WBRT-SIB. Fifteen patients had a KI ≤ 80%, median number of BMs was 8 for the entire series and 15 patients (43%) had ≥ 10 BMs. Brainstem metastases and limited leptomeningeal disease were no exclusion criteria to consider a WBRT-SIB. However, only 37% of the patients had a NSCLC diagnosis; patients with SCLC and melanoma were also considered for this treatment option.

**Table 3:** Recent studies regarding whole brain radiotherapy + simultaneous integrated boost

Study (Year)	Nr. of patients	Primary	Nr. of BMs	Therapy	Dose/ Fractionation	Outcome
Zhang et al. (2023) Retrospective [9]	*165*	NSCLC (n = 79) SCLC (n = 63) Breast (n = 15) Other (n = 8)	*NR*	*WBRT-SIB (n* = *82)* *WBRT (n* = *43)*	WBRT-SIB: 25–37.5Gy/10–20 Fr, SIB 35–52.5Gy/10–20 Fr	WBRT + SIB group longer local control and iPFS (P = 0.006 and 0.042, respectively); no improvement in OS
Dong et al (2023) Retrospective [10]	*90*	*NSCLC*	*1 (n* = *34)* > *1 (n* = *46)*	*WBRT-SIB (n* = *42)* *SRS (n* = *48)*	WBRT-SIB: 30 Gy/10 Fr, SIB 45Gy/10 Fr SRS: 16–24 Gy	longer median iPFS WBRT-SIB (20 vs 12 mo, P = 0.0069); similar median local control and OS 32 vs 28 mo, NS
Rades et al (2023) Retrospective [11]	*378*	*NSCLC* *Breast* *SCLC* *Other*	*NR*	WBRT (n = 275) WBRT-SIB (n = 103)	WBRT-SIB: 35 Gy/14 Fr, SIB 42Gy/14 Fr or 36 Gy/18 Fr, SIB 45Gy/18 Fr	WBRT-SIB: improved intracranial controll but similiar OS
Ma et. al (2022) Retrospective [12]	16	NSCLC	0–4 (n = 10) 4–10 (n = 4) > 10 (n = 2)	WBRT-SIB + Apatinib	WBRT-SIB: 37.5 Gy/15 Fr, SIB of 49.5–52.5 Gy/15 Fr	Median iPFS 16,5 months, median OS 26 months, 2 patients had grade 3 hypertension and oral mucositis, respectively
Popp et al (2022) Dosimetric [13]	30	Melanoma Lung Breast GI	3–11 BM or Resection cavities	HA-WBRT-SIB	WBRT-SIB: 30Gy /12 Fr, SIB 36-51Gy/ 12 Fr	Lower mean dose to hippocampus in hippocampal blocking compared to conventional method: 8.8 Gy vs. 10 Gy (p = 0.003)
Zhai et. al (2021) Retrospective [14]	61	NSCLC	≤ 3 (n = 23) > 3 (n = 38)	Osirmetinib mono (n = 40) WBRT + Osirmetinib (n = 14) WBRT-SIB + Osirmetinib (n = 5) SRS + Osirmetinib (n = 2)	WBRT 30 Gy/ 3 Gy WBRT-SIB 30 Gy /10 Fr, SIB 40–50 Gy/10 Fr	No difference in iPFS, median OS 29 mo Osimertinib + RT vs. 26 mo osimertinib mono, 3 patients had leukoencephalopathy group RT + Osirmet. grade ≤ 3
Lin et al. (2021) Retrospective [15]	72	NSCLC	1 (n = 28) ≥ 2 (n = 44)	WBRT-SIB (n = 37) WBRT + SRS (n = 35)	WBRT-SIB: 30 Gy/10 Fr, SIB 45G/10Fr WBRT + SRS 30Gy/10 Fr + 16–24 Gy	Median iPFS longer in WBRT + SIB (9.1 vs. 5.9 mo, P = 0.001) than in WBRT + SRS group
Du et. al (2021) Retrospective [16]	144	NSCLC	1 (n = 36) > 1 (n = 108)	WBRT (n = 77) WBRT-SEB (n = 38) WBRT-SIB (n = 28)	WBRT 40Gy/20 Fr WBRT-SEB 40 Gy/ 20 Fr, BMs 16–26 Gy/ 6–13 Fr WBRT-SIB: 36–41,4 Gy/20–24 Fr, SIB of 56–62,4 Gy/20–24 Fr	Median OS significantly longer SIB-IMRT group (14 mo) than in WBRT group (7 mo) p < 0.001 and WBRT + SEB group (11 mo) p = 0.037
Westover et al (2020) Phase II trial [17]	50	Lung (n = 39) Breast (n = 5) Other (n = 6)	1–3 (n = 18) 4–6 (n = 24) 7–8 (n = 8)	WBRT-SIB (n = 50)	WBRT-SIB: 20Gy/10 Fr, SIB 40Gy/10 Fr	Median PFS 2,9 mo, median OS 9 mo, 1-y local relapse 8,8%, 1-y intracranial relapse 21%
Qing et al. (2020) Retrospective [18]	52	NSCLC	1–2 (n = 24) ≥ 3 (n = 28)	WBRT-SEB (n = 30) WBRT-SIB (n = 22)	WBRT-SEB 30 Gy/ 10 Fr, BMs 12 Gy/ 3 Fr WBRT-SIB 30Gy/10 Fr, SIB 40 Gy/10 Fr	WBRT + SEB less neurocognitive impairment and better survival than WBRT + SIB (15 mo vs. 10 mo), especially for male patients, age < 60 years with 1–2 BM
Dong et al. (2018) Retrospective [19]	46	NSCLC	1 (n = 15) 2–3 (n = 16) ≥ 4 (n = 15)	WBRT-SIB	WBRT 37.5 Gy/15 Fr, SIB 52.5 Gy/15 Fr	Median OS and PFS were 20 mo and 11 mo, respectively

iPFS (intracranial progression free survival), OS (overall survival), NR (not reported), WBRT-SIB (whole brain radiotherapy – simultaneous integrated boost), WBRT-SEB (whole brain radiotherapy – sequential boost), HA-WBRT (hippocampus avoidance whole brain radiotherapy), SRS (stereotactic radiosurgery), BM (brain metastases), NS (not significant), NSCLC (non-small cell lung cancer), GI (gastrointestinal), Fr (fractions)

Principally, WBRT-SIB dose was 30 Gy in 10 fractions for WBRT with a simultaneously applied Boost up to 40–52 Gy in other series [9, 10, 14, 15, 19]. However, fractionation and dose are inhomogeneous, even though the treatment was delivered in 10 up to 15 fractions. In our series, 35 Gy (WBRT)/42 Gy (SIB)/14 fractions was mostly used.

In the current literature, iPFS ranged between 3 and 20 months, often about 9–14 months, and OS ranged between 10 and 32 months, often about 20 months. In our series, median iPFS was not reached and the 1-year iPFS was 75%. The 1-year OS was about 25% (median < 9 months). Our results are in line with the current literature emphasizing a benefit of dose escalation also in multiple or large brain metastases by achieving good local and intracranial control. The possible advantages of local dose escalation include the following therapeutic goals: Firstly, significant tumor regression can be achieved in large metastases that would otherwise lead to irreversible symptoms. Furthermore, significantly better intracranial control can be achieved in patients undergoing systemic therapy with an unclear response probability due to insufficient intracranial availability of the chemotherapy or immune checkpoint inhibition (ICI) therapy used. On the other hand, a good patient selection and further findings regarding prognostic factors in an apparently unfavorable population could help to define subgroups due to the present low OS.

WBRT-SIB was reported to be well-tolerated with occasionally grade 3 toxicities [9–12, 14–19]. A hippocampus-sparing technique has been developed over time because of significant neurocognitive decline following WBRT [25]. Radiation planning with hippocampus-sparing is complicated and requires high expertise [13] in case of WBRT SIB in patients with up to 10 metastases. In the real life, patients present with > 10 BMs, grown symptomatic and they need a quick treatment administration. Moreover, in our series patients had oft metastatic growth in the hippocampus region: a sparing of the hippocampi was passible only in 3 patients.

Some of the recent studies compare WBRT-SIB to other treatment options as WBRT-SEB and WBRT + SRS [14–16, 19]. Basically, contradictory results have been reported. Lin et al. demonstrated a significant benefit in terms of iPFS after WBRT-SIB vs. WBRT + SRS (9.1 vs. 5.9 months, P = 0.001). Another study by Du et al. showed a significant OS benefit in the WBRT-SIB group (14 months) vs. WBRT alone (7 months, p < 0.001) and WBRT-SEB (11 months, p = 0.037) [16]. Instead, the retrospective analysis by Quing et al. demonstrated a significant improvement in OS in the WBRT-SEB group 15 mo vs. 10mo of the WBRT-SIB group and also better-preserved cognitive functions in favor of the WBRT-SEB group (18]. At the present, there is a lack of prospective data or at least large series of patients to draw validated conclusions, and retrospective data might suggest that a form of boost to the macroscopic intracranial disease might be superior to WBRT alone in selected cases.

Recently, efforts have been done to combine last generation systemic therapies to WBRT-SIB in patients with multiple BMs also compared to standard WBRT in NSCLC patents [12, 14]. The number of treated patients is very small, and a real difference of the addition of the drug to WBRT versus a monotherapy was not detected. It could only be assumed that a combination of systemic therapy to WBRT (± SIB) in terms of tolerance is feasible.

Most of the evidence for WBRT-SIB comes from retrospective series, indicating a need for further prospective studies to better establish the efficacy and safety of this treatment approach.

The limited number of patients in these studies (around 400 in the past 5 years) underline the need for larger cohorts and longer-term follow-up to draw more robust conclusions.

## Conclusions

In summary, the use of WBRT-SIB in patients with unfavorable conditions and a substantial burden of brain metastases was explored in this study. The treatment approach involves a combination of whole brain radiotherapy and a simultaneous integrated boost to specific tumor volumes, and its effectiveness was compared with other treatment modalities in the literature. Further research, including prospective studies with larger patient cohorts, is necessary to validate and refine the findings.

## Data Availability

Data availability The data that support the findings of this study are not openly available due to reasons of sensitivity and are available from the corresponding author upon reasonable request. Data are located in controlled access data storage at University Hospital Giessen.
